# Cumulative Ambient Light Exposure Affects Outpatient Transcutaneous Bilirubinometer Readings

**DOI:** 10.3390/children12050639

**Published:** 2025-05-15

**Authors:** Emily Zhang, Tzong-Jin Wu, Mark L. Hudak, Ke Yan, Ru-Jeng Teng

**Affiliations:** 1 Human Biology, Health, and Society, College of Human Ecology, Cornell University, Ithaca, NY 14853, USA; ezz6@cornell.edu; 2 Division of Neonatology, Department of Pediatrics, Medical College of Wisconsin, Milwaukee, WI 53226, USA; twu@mcw.edu (T.-J.W.); rteng@mcw.edu (R.-J.T.); 3 Division of Neonatology, Department of Pediatrics, University of Florida Health Science Center at Jacksonville, Jacksonville, FL 32209, USA; mark.hudak@ufhealth.org; 4 Division of Bioinformatics and Quantitative Child Health, Department of Pediatrics, Medical College of Wisconsin, Milwaukee, WI 53226, USA

**Keywords:** transcutaneous bilirubinometer, neonatal jaundice, bilirubin, season, postnatal age

## Abstract

Background: We recently reported that the transcutaneous bilirubinometer (TCB) tends to underestimate the severity of neonatal jaundice (NJ). We hypothesize that the cumulative ambient light exposure contributes to the discrepancy. Objectives: This study aimed to identify factors that affect the TCB underestimation. Methods: We analyzed prospectively collected data over a twenty-month period at a level III medical facility. Neonates at risk for NJ who couldn’t secure an appointment with the primary practitioner were followed by the nursery team. Neonates who had phototherapy or forehead bruises were excluded. Concurrently collected total serum bilirubin (TSB) was determined by the diazo method. The primary endpoint was the discrepancy between TCB and the corresponding TSB (TCB-TSB). A mixed-effects model was used to assess the correlation between (TCB-TSB) and potential contributors, including visit age (in hours), gestational age (GA), sex, TSB, season, birth weight, and race. Results: There were 795 visits for 559 neonates, including 341 males, 179 white, 235 black, 103 Hispanic, 41 Asian, and one unrecorded race. The TSB ranged between 1.8 and 33.9 mg/dL. The (TCB-TSB) ranged between −20.0 and 6.4 mg/dL. The median GA and birth weight were 38.7 weeks and 3214.5 g. The visits occurred between 48 and 381 h of age. 133, 148, 132, and 146 visits were in Spring, Summer, Autumn, and Winter, respectively. Fifty-four neonates (9.7%) were admitted for management. 500 sternum TCB readings were also collected from 350 neonates together with the corresponding forehead TCBs. We found that the forehead (TCB-TSB) was significantly less in winter than in spring and summer (*p* = 0.0014 and 0.0003, respectively). There was a negative correlation between forehead (TCB-TSB) and visit age in hours (*p* = 0.0006). After adjusting for visit age and season, the (TCB-TSB) is significantly correlated with TSB (*p* < 0.0001). Similar findings were also seen in the sternum (TCB-TSB) except for the season (*p* = 0.0808). Conclusions: Cumulative ambient light exposure and the severity of NJ may contribute to (TCB-TSB).

## 1. Introduction

Neonatal jaundice (NJ) is commonly seen in neonates, with less than 10% requiring phototherapy or exchange transfusion [1]. Nowadays, most neonates are sent home before 72 h of life, while jaundice levels are still rising [2]. Another concern is that the adequacy of oral intake is usually not fully established before nursery discharge. The American Academy of Pediatrics (AAP) recommends that neonates be evaluated by medical professionals within 48 h of discharge from the nursery to assess weight change and jaundice [3]. Total serum bilirubin (TSB) using the diazo method is considered the standard method to determine the severity of NJ. However, obtaining the TSB results might take hours, which has burdened the outpatient services. Transcutaneous bilirubinometers (TCB) have been introduced to clinical practice since 1980 [4]. They can provide readings within minutes. Multiple studies have shown TCB as a useful screening tool for managing NJ [5]. The convenience has led AAP to recommend incorporating TCB into NJ management [6].

To serve as a screening tool, we expect TCB will not underestimate the severity of NJ, as bilirubin-induced neurologic damage can lead to long-term developmental delay, cognitive impairment, and behavioral disorders [7]. We recently reported that TCB obtained by BiliChek^®^ (Respironics, Marietta, GA, USA; presently owned by Philips Healthcare) tends to underestimate the severity of NJ, especially when TSB levels are closer to the therapeutic threshold [8]. In that study, we also observed that the sternum performs better than the forehead for TCB measurements, presumably due to less ambient light exposure. Bilirubin is degraded by photoisomerization by absorbing the blue-green light [9]. It is possible that a higher amount of subcutaneous bilirubin accumulation results in more light-induced degradation that affects the TCB readings. Another important finding in that report is that TCB reading at the sternum is less underestimated than at the forehead, which can also be explained by less light exposure [8]. With these observations, we hypothesize that cumulative light exposure contributes to the TCB underestimation.

Factors affecting light absorption include skin color and tone [10,11], skin site [8], cumulative exposure time, season, and race [12]. After introducing TCB to our practice, we prospectively recorded predetermined data, including the mother’s reported race, from neonates followed by our nursery team as a quality improvement and quality assurance activity. TCB and concomitant TSB were collected, and the (TCB-TSB) was calculated accordingly for each visit. Data from the record was analyzed retrospectively. We identified that visit age, season, and TSB were significantly associated with the (TCB-TSB) value.

## 2. Materials and Methods

### 2.1. Background Information

Infants born at the Shands Hospital of the University of Florida at Jacksonville between 1 October 2004, and 2 August 2006, with concerns of enteral intake adequacy or at risk of NJ, were recommended to come back to the nursery within one or two days if an appointment with the primary practitioner was unavailable. As Jacksonville is the largest city by area in Florida, USA, we offered this service as an alternative for parents during the early stage of implementing the 2004 AAP guidelines [3]. Neonates who were already being treated by phototherapy or with forehead bruises were excluded. The hospital policy did not allow the nursery personnel to follow neonates discharged from the neonatal intensive care unit. The race was recorded according to the mother’s report. Gestational age (GA), birth weight (BBW), sex, race, visit age in hours, visit date, TSB, TCB, and disposition were recorded prospectively for each visit as a quality assurance/improvement (QA/AI) project for introducing the TCB to the hospital practice. The physicians assigned to the nursery determined the hospitalization according to the hour-specific thresholds and risk factors published in the 2004 AAP guidelines [3]. The etiology of the NJ was not recorded, as a full workup was not performed unless the neonate was hospitalized. The only identifier was the temporal medical record number attached to the mother’s medical record, which became inactive and inaccessible automatically after three years. A number was given to each neonate sequentially for our records. The Institutional Review Board of the University of Florida Health Science Center at Jacksonville granted an expedited approval (UFHSC-Jax-2004-08-01) on 1 August 2004. It waived the informed consent, considering the nature of the QA/QI.

### 2.2. Bilirubin Measurements and Clinical Disposition

Forehead TCB was obtained with the ceiling light off to minimize the influence of ambient light [13]. BiliChek^®^, provided by the Children’s Miracle Network as a gift to the hospital, was used for this study. Five allied health professionals of the newborn nursery were trained to operate the instrument to cover the service. A disposable calibration tip was applied to the detector for each neonate to avoid infection. The internal algorithm provided an average of five successful readings as the TCB readout. Blood was collected for TSB after the TCB readings and immediately shielded from light for shipment to the central laboratory within 15 min for processing. The TSB levels were determined by the classical diazo method of Jendrassik-Grof (Modular P800, Roche). The machine was routinely calibrated twice daily against the standards provided by the manufacturer. Technicians from the central laboratory reported directly to the nursery for TSB results by phone to expedite clinical dispositions. The BiliChek^®^ was returned to the manufacturer once for calibration, and no faults were detected.

### 2.3. Statistical Analysis

Data were analyzed retrospectively after de-identification in accordance with IRB guidelines. Descriptive statistics were computed for all the variables, including medians and ranges for continuous variables, and frequency counts with percentages for categorical variables. We focused mainly on the forehead TCB readings, with 295 more paired data points and better power than the sternum TCB. The Bland-Altman plot evaluates the agreement between forehead TCB readings and TSB values. To account for multiple visits per neonate, a mixed-effects model was used to assess the correlation between (TCB-TSB) and various factors, including visit age (in hours), GA, sex, TSB, season, BBW, and race. SAS 9.4 (SAS Institute Inc., Cary, NC, USA, 2016) and IBM SPSS Statistics 29 (IBM Corp., Armonk, NY, USA) were used for all the analyses. A *p*-value < 0.05 was considered statistically significant. The Bland-Altman plot and the folded empirical cumulative distribution (mountain) plot were generated by the MedCalc^®^ Statistical Software version 23.06 (MedCalc Software Ltd., Ostend, Belgium; https://www.medcalc.org (accessed on 15 March 2025).

## 3. Results

### 3.1. Demographics

**Figure 1:**
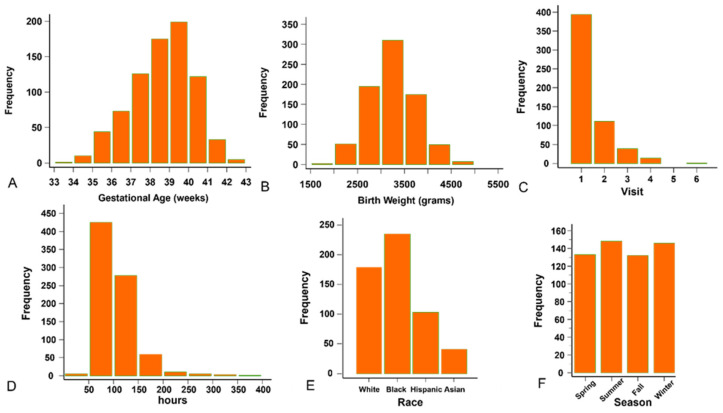
The background information of the enrollees. (**A**) Distribution of the gestational age. (**B**) Distribution of the birth weight. (**C**) Distribution of the visits. (**D**) Distribution of the age at the time of visit. (**E**) Racial distribution. (**F**) Number of neonates in each season.

Our records revealed 559 (341 males, 61.0%) neonates and 795 visits. The median gestational age was 38.7 weeks (range 33–42.3, Figure 1A), and median birthweight was 3214.5 g (range 1910–4958, Figure 1B). The number of visits per infant ranged between 1 and 6 (Figure 1C). The median visit age was 96 h (range 48–381, Figure 1D). The racial distribution (Figure 1E) was 179 Caucasian (32.0%), 235 African American (42.0%), 103 Hispanic (18.4%), 41 Asian (7.3%), and one without record (0.3%). 133 (23.8%), 148 (26.5%), 132 (23.6%), and 146 (26.1%) neonates were visited in Spring, Summer, Autumn, and Winter (Figure 1F), with 200, 207, 184, and 204 total visits, respectively. Five hundred sets of forehead and sternum TCB readings and TSB were collected from 350 neonates.

Fifty-four of the 559 (9.7%) neonates were admitted for phototherapy or blood exchange transfusion as judged by the 2004 AAP guidelines [3]. Ten of the 559 neonates (1.8%) were less than 35 weeks’ gestation, and among them, three were admitted for treatment.

### 3.2. The Agreement Between the Forehead TCB and TSB

**Figure 2:**
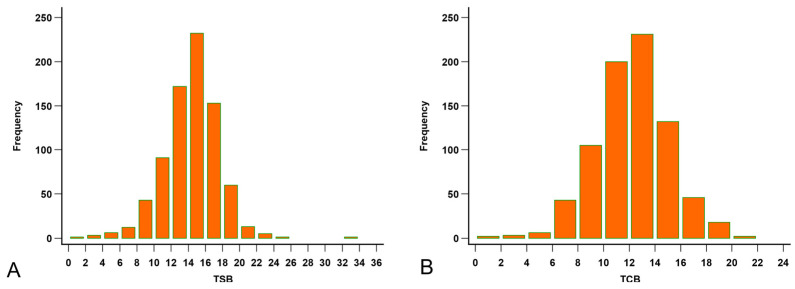
Distribution of the bilirubin levels. (**A**) Distribution of the total serum bilirubin (TSB, mg/dL). (**B**) Distribution of the forehead transcutaneous bilirubin (TCB) readings.

All 795 visits had TSB results, but seven corresponding forehead TCBs were not recorded. The medians of TSB and the forehead TCB were 14.6 mg/dL (range 1.8–33.9; Figure 2A) and 12.2 mg/dL (range 1.2–21.8; Figure 2B), respectively.

**Figure 3:**
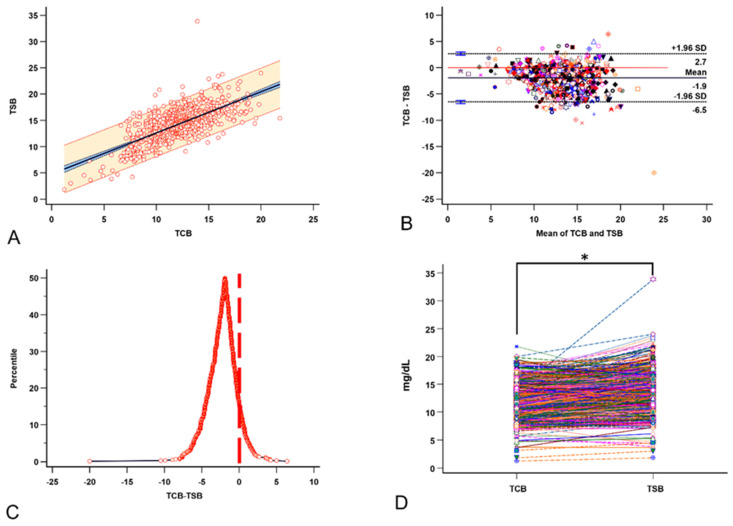
The relationships between TCB and the corresponding TSB. (**A**) A good linear relationship exists between TCB and TSB. (**B**) The Bland-Altman plot for multiple observations per individual between TCB and TSB. (**C**) The mountain plot distribution of the (TCB-TSB) shows that TCB underestimates TSB. (**D**) The paired *t*-test shows that TCB significantly underestimates the severity of neonatal jaundice (NJ). Each color, symbol, and line represents the data from the same visit. * *p* < 0.05 by paired *t*-test.

A mixed-effects model indicates a significant positive correlation between TCB and TSB; each unit increase in TCB leads to an estimated increase of 0.7 in TSB (*p* < 0.0001, Figure 3A). The Bland-Altman plot showed that the TCB readings tend to be lower than the concurrent TSB levels (median −1.9, range −20 to −6.4, Figure 3B). The TCB underestimation was also demonstrated in the mountain plot of the (TCB-TSB) distribution (Figure 3C). The paired comparison showed a significant difference between the two (Z = −20.2, *p* < 0.0001, Figure 3D).

### 3.3. Factors That Affect the Forehead (TCB-TSB) Value

**Figure 4:**
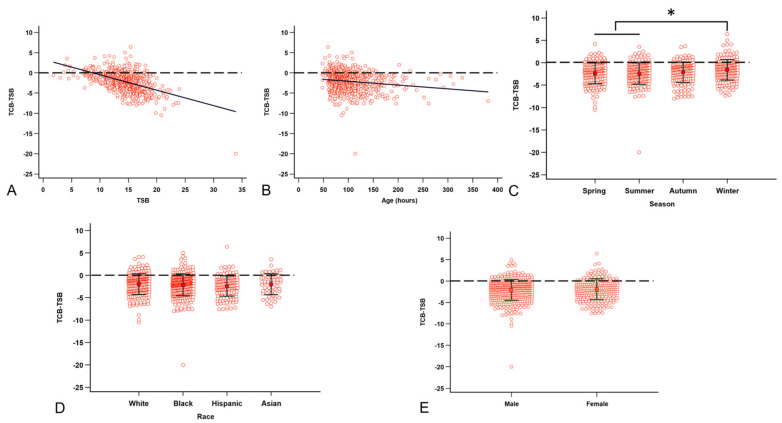
Factors that are correlated with the TCB underestimation of NJ. (**A**) The higher the TSB, the higher the (TCB-TSB) discrepancy. (**B**) Visiting age correlated with the (TCB-TSB) discrepancy. (**C**) The (TCB-TSB) underestimation is less in Winter than in Spring and Summer. (**D**) Race does not affect (TCB-TSB). (**E**) Sex does not affect (TCB-TSB). The dashed line denotes TCB equal to TSB. * *p* < 0.05 by ANOVA with Tukey post-hoc test.

Factors that are associated with the (TCB-TSB) include sex, TSB, race, visit age, GA, BBW, and season. Under univariable analysis, we detected that TSB (Figure 4A), visit age (Figure 4B), and season (Figure 4C) are significantly associated with the (TCB-TSB) values. Both visit age (*p* = 0.0006) and the TSB (*p* < 0.0001) were negatively associated with (TCB-TSB). Compared to other seasons, Winter had a significantly lower underestimation than Spring and Summer (*p* = 0.0014 and *p* = 0.0003, respectively). There was a trend of less underestimation in Winter than in Autumn (*p* = 0.078). We did not see a difference in the underestimation by race (Figure 4D) and sex (Figure 4E). We analyzed the effect of race on (TCB-TSB) by season and still saw no difference. There is no correlation between (TCB-TSB) and BBW (*p* = 0.4) or between (TCB-TSB) and GA (*p* = 0.22). In the multivariable models, the TSB remained significantly correlated with the (TCB-TSB) after adjusting for visit age and season (*p* < 0.0001). We performed the multicollinearity diagnosis and confirmed that the model is stable. To rule out the concern that high TSB levels may lead to unrecordable TCB, we performed analyses by removing either the visit with TSB of 33.9 mg/dL or all visits with TSB above 20.0 mg/dL. The removal of visits with high TSB values results in a better model with even lower *p*-values.

### 3.4. Factors That Affect the Sternum (TCB-TSB) Value

We have previously reported that the sternum is a better site for TCB reading [8]. We use the same model to analyze factors that might be associated with the sternum (TCB-TSB). As Florida is a southern state in the USA with warmer temperatures and most households have a thermostat, neonates are usually not swaddled continuously. However, the sternum is still less exposed to ambient light than the forehead. This might explain why sternum TCB is significantly closer to the corresponding TSB than the forehead TCB (−1.92 ± 2.23 vs. −2.63 ± 2.30; *n* = 471, Z = 7.21, *p* < 0.0001). Both visit age (*p* < 0.0001) and the TSB (*p* < 0.0001) were negatively associated with the sternum (TCB-TSB). We did not observe an association between season and the sternum TCB in the mixed-effects model.

## 4. Discussion

Although kernicterus and bilirubin-induced brain damage occur in only 1~3 in 100,000 live births in developed countries, it can be a significant medicolegal issue and a hefty burden on the family [14,15]. Severe NJ can account for 3% of all admissions in some developing and underdeveloped countries [16]. The global health impact of severe NJ has led to the universal screening policy. AAP recommended that its members use TCB or TSB to make decisions according to the hour-specific, risk-factor-adjusted nomograms [3,6]. The increased workload from waiting for the TSB results, after implementing the 2004 AAP guidelines [3], has encouraged clinicians to adopt TCB as the NJ screening tool.

Bilichek^®^, JM-103, and JM-105 are NJ’s most extensively studied TCBs [5]. Direct comparisons between Bilichek, JM-101, and JM-103 are unavailable, but available data indicate a good correlation between TCB and TSB. However, both underestimation [17] and overestimation [18] of the severity of NJ have been reported. Possible explanations include that different methods have been used for TSB measurements in other studies [5], and different internal algorithms used by each TCB. The diazo method is considered the gold standard [19]. A suitable screening tool must be highly sensitive without underestimating NJ severity. We previously reported that the TCB obtained by our Bilichek underestimated the seriousness of NJ, with more than 50% of neonates who qualified for hospital admission being sent home if the companion TSB was not obtained [8]. This finding should be considered before adopting TCB for outpatient NJ screening.

Our data in this study (Figure 3A,B) corroborate other reports showing a good correlation between TCB and TSB [5]. However, we cannot ignore that TCB readings are significantly lower than the corresponding TSB readings in our practice (Figure 3C,D). Univariable analysis showed that TSB, visit age, and season are significantly associated with (TCB-TSB) values. The higher the TSB reading, the more significant the underestimation of the severity of the NJ (Figure 4A). The older visit age (Figure 4B) and visits in spring and summer (Figure 4C) are also associated with more significant TCB underestimation than in winter. Unfortunately, we did not collect skin color and tone during this project. However, race (Figure 4C), sex (Figure 4D), GA, and BBW do not affect the (TCB-TSB) in our study. The association of TSB, visit age, and season with (TCB-TSB) persists in the multivariable backward regression after controlling for all potential variables, confirming our findings from the univariable analyses.

Different TCB models on the market use different algorithms [5]. Our findings may not represent all BiliChek^®^ models in use, as we used the original model, which may have undergone continuous upgrades. However, analyzing data using repeated measures increases the power of the analysis. Ambient light has been postulated to explain the differences between TCB and TSB [20]. The cumulative light exposure to the forehead can explain the association between visit age and (TCB-TSB). The cold weather and less sunlight in Florida also reasonably explain why (TCB-TSB) is lower in winter. We are intrigued by the significant correlation between TSB and (TCB-TSB). A possible explanation is the light absorption by the subcutaneous bilirubin deposition. Our findings support the hypothesis that cumulative light exposure/absorption contributes to the (TCB-TSB) value. The influence of ambient light is why BiliEclipse, which can be applied to the forehead to shield the ambient light, is promoted by the Bilichek manufacturer.

In a previous report, race, gestational age (GA), and body weight (BW) were suggested as factors affecting TCB accuracy [21]. Bilichek^®^ has been promoted due to its unique advantage of isolating bilirubin’s light absorption from other factors such as hemoglobin, melanin, and dermal thickness [21]. In the present study, race, GA, or BW associated with the forehead (TCB-TSB), supporting the device’s widespread clinical applicability.

Our prior report raised concerns about TCB underestimating the severity of NJ [8]. Others have reported similar findings [22]. Although the chance of kernicterus or bilirubin-induced brain damage is extremely low [14,15], we cannot ignore the potential liability. For example, one of our neonates was admitted for blood exchange transfusion for a TSB level of 33.9 mg/dL at 114 h of life, but the corresponding TCB reading was 13.9 mg/dL. Without obtaining the TSB, we will probably manage the neonate inadequately. We specifically excluded neonates treated by phototherapy, under the assumption that the treatment can exaggerate the TCB underestimation [23]. The association between forehead (TCB-TSB) and TSB, age of visit, and season has not been examined before. A plausible explanation is the cumulative ambient light exposure. Although we did not see the association between sternum (TCB-TSB) and season, the trend (*p* = 0.0808) might indicate less power due to fewer data points, or the fact that the sternum receives less ambient light than the forehead.

There are limitations to this study. First, we did not attempt to identify the etiology of the NJ as the service was not established to substitute for the role of the primary physician. Second, our original QA/QI project did not record other risk factors such as blood type, hemolytic disease, trauma, breastfeeding, and family history of NJ that may be associated with NJ. Third, the skin color and tone were not recorded. Fourth, the mothers determined the race of their neonates, and mixed-race is common in the area. The study aimed to examine whether cumulative ambient light exposure is associated with the underestimation of NJ by forehead TCB readings. Undoubtedly, TCB has a good correlation with TSB [24]. Our study findings suggest that TCB readings must be validated against TSB before being adopted as an NJ management tool.

## 5. Conclusions

TCB is a convenient tool for NJ management, but its underestimation of the severity of the NJ needs our attention. The mixed model indicates that race, GA, BBW, and sex do not affect the differences between TCB and TSB. Visit age, season, and TSB level are three factors that affect the extent of underestimation by the forehead TCB. Although we cannot prove that cumulative ambient light exposure causes the underestimation by TCB, we would like to caution pediatricians about this association when using TCB as the screening tool for outpatient NJ management.

## Data Availability

Raw data are available upon formal request.

## Abbreviations

AAP	American Academy of Pediatrics
GA	Gestational age
NJ	Neonatal jaundice
TCB	Transcutaneous bilirubinometer
TSB	Total serum bilirubin
LD	Linear dichroism
